# Plasma Epstein–Barr Viral Deoxyribonucleic Acid Predicts Worse Outcomes in Pediatric Nonmetastatic Nasopharyngeal Carcinoma Patients: An Observational Study of 89 Cases in an Endemic Area

**DOI:** 10.1097/MD.0000000000001945

**Published:** 2015-12-18

**Authors:** Ting Shen, Lin-Quan Tang, Wei-Guang Gu, Dong-Hua Luo, Qiu-Yan Chen, Pei-Jing Li, Dong-Mei Mai, Hai-Qiang Mai, Hao-Yuan Mo

**Affiliations:** From the State Key Laboratory of Oncology in South China, Collaborative Innovation Center for Cancer Medicine (TS, L-QT, D-HL, Q-YC, P-JL, D-MM, H-QM, H-YM); Department of Nasopharyngeal Carcinoma, Sun Yat-sen University Cancer Center, Guangzhou (TS, L-QT, D-HL, Q-YC, P-JL, D-MM, H-QM, H-YM); and Department of Oncology, The People's Hospital of Nanhai District, Foshan, The People's Republic of China (TS, W-GG).

## Abstract

To evaluate the clinical significance of pretreatment levels of plasma Epstein–Barr virus DNA (pEBV DNA) on prognoses in pediatric nasopharyngeal carcinoma (NPC) patients.

Eighty-nine patients aged 21 years old or younger with nonmetastatic NPC were evaluated to determine the effect of pEBV DNA levels on progression-free survival (PFS), distant metastasis-free survival (DMFS), and overall survival (OS). Survival probabilities in patient groups that were segregated by clinical stage or pEBV DNA load (low or high) were compared.

The median pretreatment concentrations of pEBV DNA were 3440 copies/mL in 35 patients with stage III disease and 14,900 copies/mL in 50 patients with stage IV disease (*P* = 0.059). The median concentration of pEBV DNA was 34,500 copies/mL in 17 patients with relapse, which was higher than the concentration in 72 patients without relapse, who had a median level of 4985 copies/mL (*P* = 0.057). Further study showed that pretreatment pEBV DNA load was an independent prognostic indicator in pediatric NPC patients. High pEBV DNA was associated with adverse clinical outcomes, including PFS [3-year PFS rate = 80.5% versus 95.8%, hazard ratio (HR) = 5.00, 95% confidence interval (CI) = 1.00–25.00; *P* = 0.050], DMFS (3-year DMFS rate = 80.5% versus 95.8%, HR = 5.20, 95% CI = 1.04–26.00; *P* = 0.045), and OS (3-year OS rate = 82.9% versus 95.8%, HR = 5.41, 95% CI = 1.08–27.22; *P* = 0.040).

Pretreatment pEBV DNA load was an independent prognostic indicator for PFS, DMFS, and OS in pediatric patients with NPC. Prospective studies, however, are needed to validate these results.

## Abstract 2

Supplemental Digital Content is available in the text

## BACKGROUND

Nasopharyngeal carcinoma (NPC) is an uncommon malignancy in children and adolescents in both low-incidence and endemic areas. The incidence of pediatric NPC varies greatly according to ethnic and geographic factors.^1–3^ In high-incidence areas, including southern China, most NPC patients are diagnosed in their fourth or fifth decade, and childhood and adolescent patients account for less than 1% of all patients.^2–4^ A bimodal age distribution with a second minor early peak corresponding to patients under 20 years of age, however, exists in low- or intermediate-risk regions, where children and adolescents account for 5% to 10% of cases.^5,6^ Because NPC is extremely rare in the pediatric population and there is a paucity of data regarding these patients, the treatment modalities for NPC have generally followed those established for adult patients. Compared with their adult counterparts, childhood and adolescent patients, however, display some characteristics that may differ vastly from those observed in adults. The majority of tumors in young patients are of the nonkeratinizing, undifferentiated subtype, whereas children and adolescents are more likely to have advanced locoregional disease at the first diagnosis, with a higher prevalence of distal metastasis. They generally have a significantly better chance of survival if the tumor, however, is more closely associated with the Epstein–Barr virus load (EBV).^3,7–9^ This prompted us to determine and evaluate prognostic factors tailored to pediatric patients.

Plasma EBV DNA (pEBV DNA) is one of the most well-recognized biomarkers for NPC.^10,11^ A cumulating body of data suggests that pEBV DNA is a useful tool to supplement the tumor, node, metastases (TNM) system used for prognostication in NPC and also for monitoring the progress of the disease.^11–13^ It is interesting that although pEBV DNA load predicts overall survival, it seems to be a better prognosticator for distant metastatic recurrence in NPC.^13,14^ This may have even greater prognostic importance when the pattern of failure is dominated by distant failure.^11^ To our knowledge, the issue of whether treatment outcomes can be predicted by pretreatment levels of pEBV DNA in young NPC patients has not been addressed, especially in endemic areas. Given this observation, it is logical to expect that pretreatment pEBV DNA levels have an effect on prognostication. Therefore, we performed a retrospective review of pediatric patients with NPC who were treated at our institution to investigate the association between pEBV DNA values and tumor stages and disease outcomes in this group, thereby providing information useful for the treatment of children and adolescents.

## PATIENTS AND METHODS

### Patients

A retrospective review was performed that included all NPC patients initially diagnosed at 21 years of age or less who were treated in our center between January 2007 and December 2011. Patients with distant metastases were not included in the study. Other cases, such as those with incomplete medical records or patients who were lost during follow-up, were also excluded. A total of 89 patients who received definitive radiotherapy alone or chemoradiotherapy, depending on the stage of disease, were included as the subjects of this study. The hospital records for each patient were reviewed for demographic and clinical data, including age, sex, World Health Organization (WHO) pathologic classification, clinical tumor stage, the level of pEBV DNA, and the radiation technology and treatment modality used. The clinical tumor stage of each patient was restaged according to the seventh International Union against Cancer/American Joint Committee on Cancer (UICC/AJCC) TNM staging manual.^15^ This study was approved by the institutional review board of Sun Yat-sen University Cancer Center, and written informed consent was obtained from all patients at recruitment.

### Plasma Epstein–Barr Virus Deoxyribonucleic Acid Measurement

As described in previous studies,^10,16,17^ patient pEBV DNA concentrations were routinely measured using quantitative polymerase chain reaction before treatment.

### Treatment

All patients received definitive radiation therapy. A total of 24 patients were treated using conventional radiotherapy, 6 patients received three-dimensional conformal radiotherapy (three-dimensional-CRT), and 59 patients were treated with intensity-modulated radiotherapy (IMRT). The median radiation dose was 70 Gy (range from 65 to 76 Gy) to the nasopharynx primary tumor and 64 Gy to the positive cervical lymph node (74 patients were positive for cervical lymph node). For patients who received IMRT, the prescription dose was 65 to 70 Gy/30 to 35 fractions to the nasopharynx gross target volume, 60 to 72 Gy/30 to 36 fractions to the gross tumor volume of lymph node (for involved lymph nodes), 60 Gy/30 fractions to the clinical target volume 1, and 54 Gy/30 fractions to the clinical target volume 2. The irradiation doses to the organs at risk were restricted to avoid exceeding their tolerance doses and to prevent sacrificing coverage of the tumor target volumes. For patients who received conventional radiotherapy or three-dimensional-CRT, the prescribed dose to the neck was 50 to 56 Gy/25 to 28 fractions for prophylaxis and 60 to 70 Gy/30 to 33 fractions for therapy.

The treatment modality was determined according to the TNM stage. In all, 37 patients (41.6%) received concurrent chemoradiotherapy, 31 patients (34.8%) were treated with induction and concurrent chemoradiotherapy, 18 patients (20.2%) received induction chemotherapy followed by radiotherapy, and 3 patients (3.4%) received radiotherapy alone, as shown in Table 1. All chemotherapy regimens were either cisplatin-based single drug or 2-drug or 3-drug combinations of 5-fluorouracil, paclitaxel or gemcitabine + cisplatin. The most commonly used induction chemotherapy regimen was 2 to 3 cycles of cisplatin/5-fluorouracil administered at 3-week intervals, whereas paclitaxel + cisplatin, gemcitabine + cisplatin or paclitaxel, and cisplatin + 5-fluorouracil were administered to a small minority of the included patients. Five to 8 cycles of single cisplatin were administered weekly as the most common regimen that was administered concurrently with radiotherapy.

**TABLE 1:**
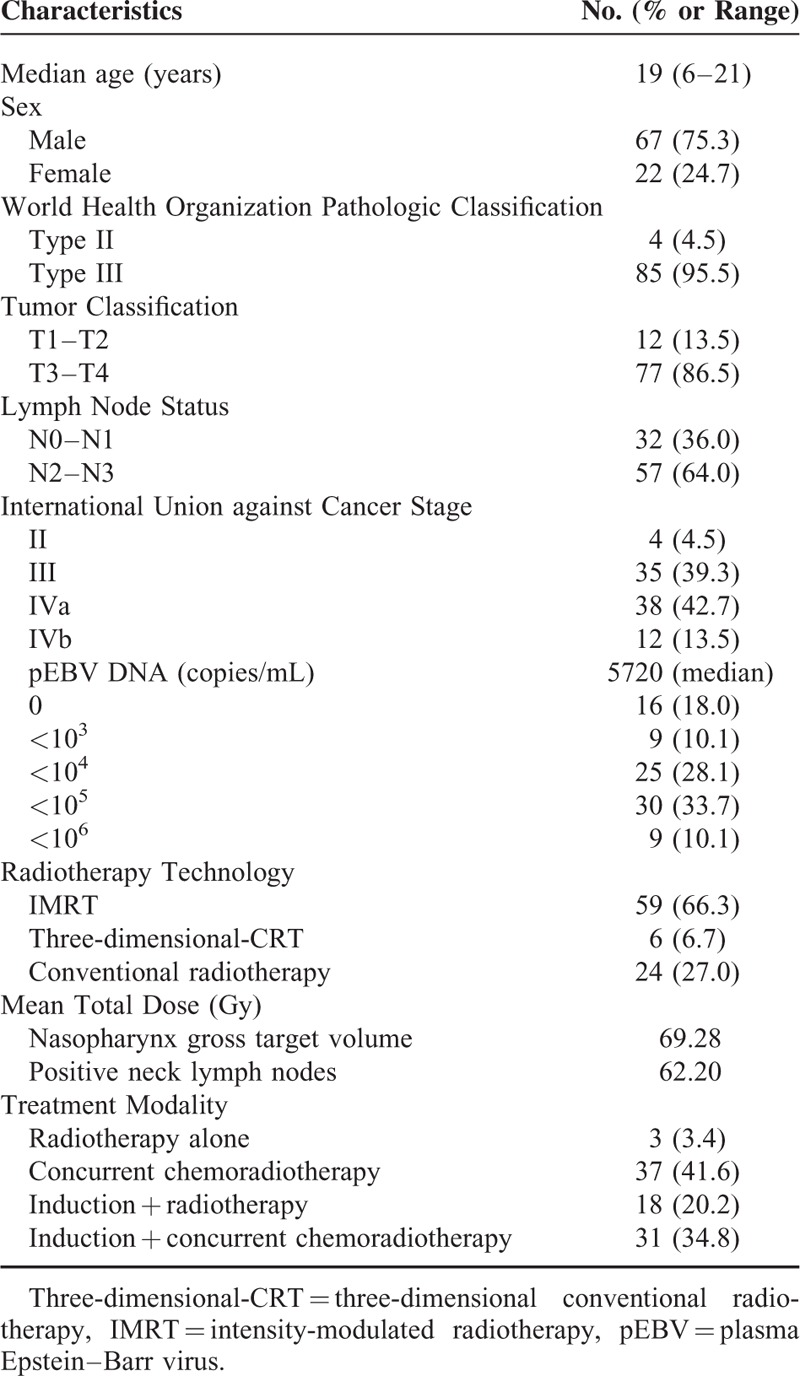
Patient Characteristics

### Clinical Outcome Assessment and Follow-Up

The first assessment of tumor responses was performed 3 months after completion of therapy. The assessment included a complete physical examination, a fiber-optic nasopharyngoscopy, magnetic resonance imaging of the head and neck, hematologic and biochemical profiles, chest radiography, abdominal ultrasonography, whole-body bone scans, and EBV serology. Then, the patients were evaluated once every 3 months during the first 3 years after therapy, once half a year for the following 2 years, and once every year thereafter. When an abnormality was identified, further investigation was arranged. The primary end point was progression-free survival (PFS), which was calculated from the date of diagnosis to the first relapse at any site, death from any cause or the date of the last follow-up visit. Overall survival (OS), as measured from the date of diagnosis to the date of death or the last follow-up, distant metastases-free survival (DMFS), as measured from the date of diagnosis to the date of distant relapse or patient censoring at the date of the last follow-up, were also assessed.

### Statistical Analysis

The Statistical Package for the Social Sciences software package (SPSS V. 17.0, SPSS, Inc., Chicago, IL) was used for data analysis. A comparison of pEBV DNA levels, the clinical UICC staging and the relationship between the pEBV DNA concentration and the relapse rate were evaluated using the Mann–Whitney *U* test. A receiver operating characteristic curve was used to determine the optimal cutoff value for pEBV DNA load that could be used to predict outcomes with the best trade-off between sensitivity and specificity1Supplemental Figure 1.
. Finally, a cutoff level of 7500 copies/mL was chosen to define low and high EBV DNA load. Multivariate analysis using a Cox regression model was performed with the following variables in the model: age, sex, pathologic type, clinical stage, radiation technology (IMRT versus concurrent radiotherapy or three-dimensional-CRT), pEBV DNA level (>7500 or ≤7500 copies/mL). Kaplan–Meier plots of OS, PFS, and DMFS were established for patients with high and low EBV DNA levels (indicated as >7500 and ≤7500 copies/mL, respectively), for patient groups with different UICC stages (stage III–stage IV), and for patients with pEBV DNA levels and UICC stages together. Log-rank tests were performed to assess the differences in survival probabilities between patient subgroups. Analyses were repeated for the following end points: PFS, OS, and DMFS. All statistical tests were 2 sided, and a *P* < 0.05 was considered to indicate statistical significance.

## RESULTS

### Patient Characteristics

The characteristics of the enrolled patients are listed in Table 1. The median age at diagnosis was 19 years old (range, 6–21 years old). The sex ratio of male/female was 3.05. Histologic types were determined according to the WHO classification system. A total of 85 patients (95.5%) had undifferentiated carcinoma (formerly WHO type III), and 4 patients had nonkeratinizing carcinoma (formerly WHO type II). Consistent with previous studies,^3,18^ 85 patients (95.5%) presented with advanced stage III or stage IV locoregional disease. The median follow-up time of our patients was 44.9 months.

### Relationship Between Epstein–Barr Virus Deoxyribonucleic Acid Concentration and Clinical Stage or Relapse

**FIGURE 1:**
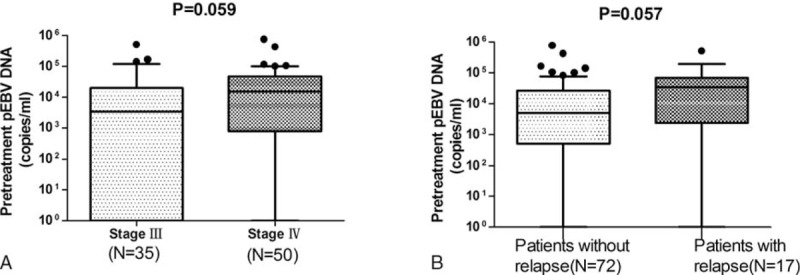
Clinical analysis of plasma EBV DNA concentrations in children and adolescents with nasopharyngeal carcinoma. Panel A shows the pretreatment plasma concentrations of EBV DNA according to the stage of disease, and panel B shows the pretreatment plasma concentrations of EBV DNA according to the presence or absence of relapse. The pretreatment plasma concentration of EBV DNA in children and adolescents with nasopharyngeal carcinoma was correlated with the clinical stage of the disease (P = 0.059) and with the occurrence of relapse (P = 0.057). In each panel, the lines inside the boxes indicate the medians, the boxes indicate the interquartile ranges, and the I-bars indicate the 10th and 90th percentiles. EBV = Epstein–Barr virus, DNA = deoxyribonucleic acid.

We detected pEBV DNA levels in plasma samples from 73 of 89 patients. Of these, pEBV DNA was undetectable in 16 patients; 10 of these had stage III disease, and 6 had stage IV disease. A total of 55 patients (61.8%) presented with a level of 1000 to 100,000 copies/mL. The median pretreatment concentration of pEBV DNA was 5720 copies/mL (interquartile range, 746–38,850 copies/mL). Of the 89 included patients, 35 presented with stage III disease, 50 presented with stage IV disease, and only 4 patients had stage II disease. The median pretreatment concentration of pEBV DNA was 3440 copies/mL (interquartile range, 0–20,000 copies/mL) and 14,900 copies/mL (interquartile range, 799–47,100 copies/mL) for patients with stage III and stage IV disease, respectively (*P* = 0.059, Figure 1A). A Spearman correlation analysis further demonstrated that pEBV DNA levels were closely correlated with the node stage and the UICC TNM tumor stage (*P* < 0.001 and *P* = 0.057, respectively; Table 2).

**TABLE 2:**
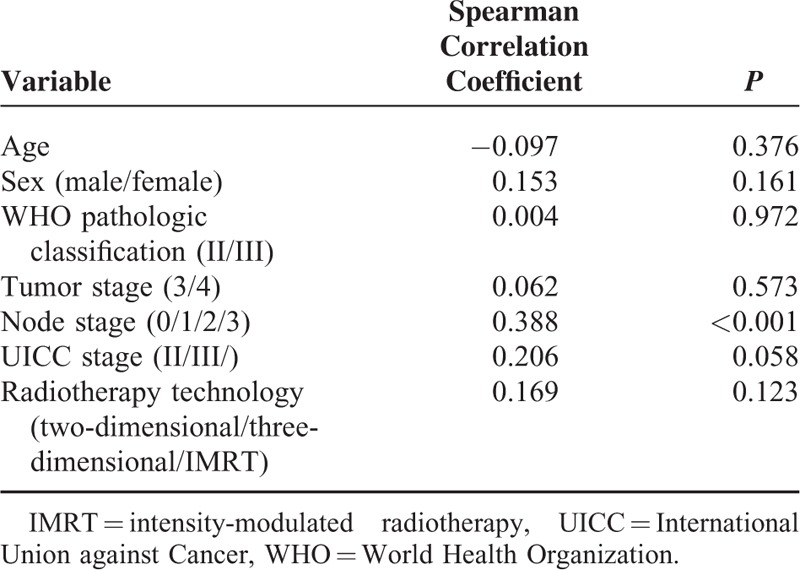
Plasma Epstein–Barr Virus Deoxyribonucleic Acid Relationships

In our study, 17 patients ended in disease relapse, including 3 patients with locoregional relapse, 13 patients with distant failure, and 1 patient with locoregional relapse and distant failure. Distant failure was the main mode of failure in pediatric patients, occurring in 82.4% of treatment failures. Patients with disease relapse had a higher level of pEBV DNA then patients without disease relapse, with a marginal level of significance (*P* = 0.057, Figure 1B). The median concentrations in these patients were 34,500 copies/mL (interquartile range, 34,700–68,000 copies/mL) and 4985 copies/mL (interquartile range, 438–23,800 copies/mL), respectively.

### Progression-Free Survival

**FIGURE 2:**
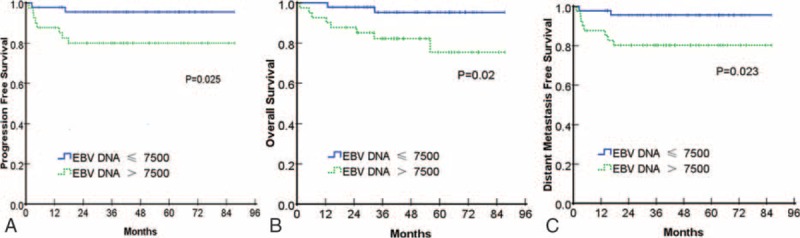
Kaplan-Meier analysis of PFS (panel A), OS (panel B) and DMFS (panel C) of patient groups according to Epstein–Barr virus DNA levels. DMFS = distant metastasis free survival, OS = overall survival, PFS = progression free survival.

Kaplan–Meier analysis of PFS was performed. The high EBV DNA group had significantly lower survival than the low EBV DNA group (3-year PFS rate = 80.5% versus 95.8%; *P* = 0.025, Figure 2A). In multivariate analyses, EBV DNA was the only independent prognostic factor for PFS with a borderline difference (HR = 5.00, 95% CI = 1.00–25.00, *P* = 0.050, Table 3).

**TABLE 3:**
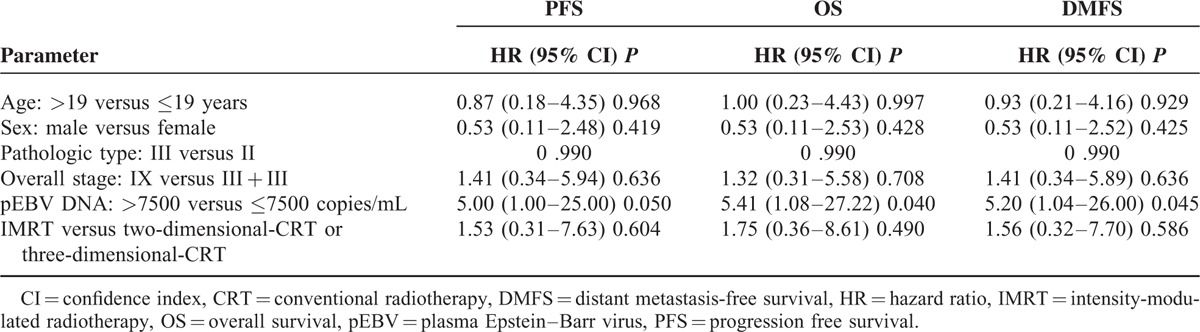
Progression Free, Overall, and Distant Metastasis-Free Survival Analyses Using a Multivariate Cox Proportional Hazards Model

The 3-year survival probabilities for different clinical characteristics (stages from stage III to stage IV) are listed in Table 4. A difference in survival rates for stage III and stage IV patients was observed, but the difference was not significant (*P* = 0.230). The 3-year survival rates for these patients were 93.9% (95% CI, 85.7%–100.0%) and 85.6% (95% CI, 75.8%–95.4%), respectively. In view of the very small number of patients with stage II disease, stage II disease was not taken into consideration in statistical analyses.

**TABLE 4:**
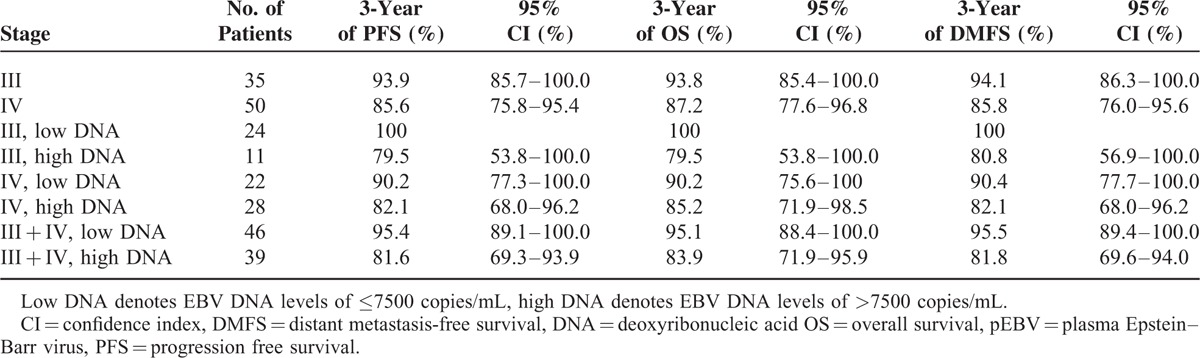
Survival Probabilities of Patients Groups With Different International Union Against Cancer Stages and With Different Epstein–Barr Virus Deoxyribonucleic Acid Levels Within International Union Against Cancer Stages

The survival probabilities in patient subgroups defined by low and high EBV DNA levels that had advanced-stage disease (stage III and IV) are also listed in Table 4. Within patients with stage III and IV diseases, high EBV DNA levels strongly predicted a lower survival rate than the rate for the low EBV DNA group (*P* = 0.047). The 3-year survival rates for the high and low EBV DNA groups containing patients with advanced-stage disease (stage III and stage IV) were 81.6% (95% CI, 69.3%–93.9%) and 95.4% (95% CI, 89.1%–100.0%), respectively.

### Overall Survival

All analyses were repeated using OS as the end point, and the same conclusions were obtained. The high EBV DNA group had a significantly lower survival rate than the low EBV DNA group (3-year OS rate = 82.9% versus 95.8%; *P* = 0.020, Figure 2B). In the multivariate analyses, pEBV DNA was the only independent prognostic factor for OS that showed a significant difference (HR = 5.41, 95% CI = 1.08–27.22, *P* = 0.040, Table 3).

### Distant Metastasis-Free Survival

Distant failure was the main mode of failure observed in our study of pediatric patients. In all, 15.7% of the cases in our study ended in distant failure, corresponding to 82.4% of all treatment failures. All analyses were repeated using DMFS as the end point, and similar conclusions were obtained. The high EBV DNA group had significantly lower survival than the low EBV DNA group (3-year DMFS rate = 80.5% versus 95.8%; *P* = 0.023, Figure 2C). In multivariate analyses, pEBV DNA was the only independent prognostic factor for DMFS (HR = 5.20, 95% CI = 1.04–26.00, *P* = 0.045, Table 3).

## DISCUSSION

In the current study, the nonkeratinizing, undifferentiated subtype of NPC was the most common histologic variant. Advanced locoregional disease, a higher rate of distant failure, and a close association with high EBV DNA levels were also observed. These findings are in accordance with findings in previous reports.^3,7–9^

To the best of our knowledge, this is the first study to evaluate prognostication according to EBV DNA load in pediatric NPC patients. The results of the current study demonstrate that pretreatment levels of pEBV DNA are correlated with the N stage and disease relapse in pediatric patients, which was in agreement with previous studies by Lo et al and Lin et al in adult NPC patients. They demonstrated that circulating pEBV DNA concentrations were correlated with tumor stage^19,20^ and the likelihood of recurrence.^20,21^

We also found that pretreatment levels of pEBV DNA are a powerful prognostic factor that is associated with progression-free, distant metastasis-free, and overall survival. High pEBV DNA levels predicted inferior outcomes for PFS, OS, and DMFS in nonmetastatic pediatric NPC patients. Patients with high pEBV DNA levels displayed a greater than 5-folds increased risk of disease progression, distant failure, and shorter overall survival than patients with low pEBV DNA levels. This effect was independent of the TNM tumor stage, radiotherapy techniques used, age, sex, and pathologic type. In patients with advanced disease (stage III and IV), patient subgroups defined by low and high pEBV DNA levels showed similar results.

Currently, treatment for pediatric patients is generally extrapolated from guidelines tailored for adult patients, but children are usually excluded from adult clinical trials because of strict age cutoffs.^22^ The optimal treatment for pediatric NPC patients, who represent a small group, is less well defined, partly because of the rarity of the disease. Previous findings related to pEBV DNA in adults might not be reliable or directly applicable in children. Our study demonstrates the prognostic value of pretreatment levels of pEBV DNA in pediatric patients, which may have a bearing on management decisions. In our study, the level of pEBV DNA was a more powerful indicator than the TNM stage. Moreover, pEBV DNA combined with the TNM stage had a refining effect on the risk stratification for overall survival. Survival probabilities showed that subgroups defined by high pEBV DNA levels and stage III disease had 3-year survival rates similar to those defined in patients with high pEBV DNA levels and stage IV disease. A subset of patients with stage IV disease also had relatively superior survival. Such observations have implications that may allow clinicians to potentially identify candidates who are eligible for aggressive therapy, thereby improving treatment outcomes. Similar results were achieved in previous studies. A study by Leung et al^11,13^ suggested a method for selecting patients for therapy intensification. According to their perspective, the therapeutic ratio could be increased by lowering the intensity of therapy in the low-risk group, whereas intensified therapy should be maintained or further augmented through the adjuvant phase in the high-risk group.^13^ A recent study showed that adjuvant chemotherapy can reduce distant failure and improve overall survival in NPC patients with persistently detectable pEBV DNA after curative radiation therapy + induction/concurrent chemotherapy.^23^ This also provides evidence that supports intensifying treatment in pediatric patients with high pEBV DNA levels.

Similar studies have been performed in untreated NPC patients who show no evidence of distant metastasis. Pretreatment pEBV DNA cutoff values have been defined differently in previous studies. Chan et al^12^ used a pretreatment cutoff value of 4000 copies/mL; however, Lin et al^20^ used a value of 1500 copies/mL. Patients in these studies, however, were not specifically young patients. Given that the patients in our study were children and adolescents with locoregionally advanced disease and a higher level of pEBV DNA concentration, previous cutoff values might not be applicable to our studies. Therefore, we used receiver operating characteristic to determine the optimal cutoff value and to determine the best trade-off between sensitivity and specificity. Taken together, our results and those of previous studies show that pretreatment levels of pEBV DNA can be a useful biomarker for predicting outcomes in NPC patients, not only in adults but also in children and adolescents.

There are some limitations to the current study, such as the relatively small sample size and short follow-up time (median follow-up time was 44.9 months). Second, measurements were recorded in a single center. Hence, a larger-scale and multicenter cohort study is warranted.

## CONCLUSIONS

In summary, we have demonstrated that the pretreatment level of pEBV DNA is a promising prognostic biomarker that is associated with progression-free, distant metastasis-free, and overall survival in pediatric patients. Pediatric patients who have a poor prognosis can be identified by measuring levels of pretreatment circulating pEBV DNA, which may indicate that they should receive intensified treatment. A prospective study to evaluate the prognostic value of pEBV DNA is needed.

## Supplementary Material

Supplemental Digital Content
