# Supplementary material for: Plasma Epstein–Barr Viral Deoxyribonucleic Acid Predicts Worse Outcomes in Pediatric Nonmetastatic Nasopharyngeal Carcinoma Patients: An Observational Study of 89 Cases in an Endemic Area
Source: Medicine (Baltimore). 2015 Dec 18;94(50):e1945. doi: 10.1097/MD.0000000000001945 (PMC5058881; doi:10.1097/MD.0000000000001945)

**Supplemental Digital Content.** Figure 1 illustrates the receiver operating characteristic (ROC) curve that determined the optimal cutoff value for EBV-DNA. A cut-off level of 7500░copies/ml was chosen to define low and high concentrations of EBV DNA, and the area under the curve is 0.689.


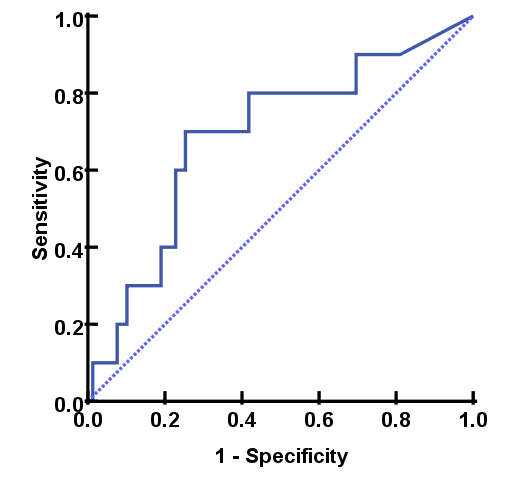


Figure 2 illustrates survival probabilities of patient groups according to the UICC stage.


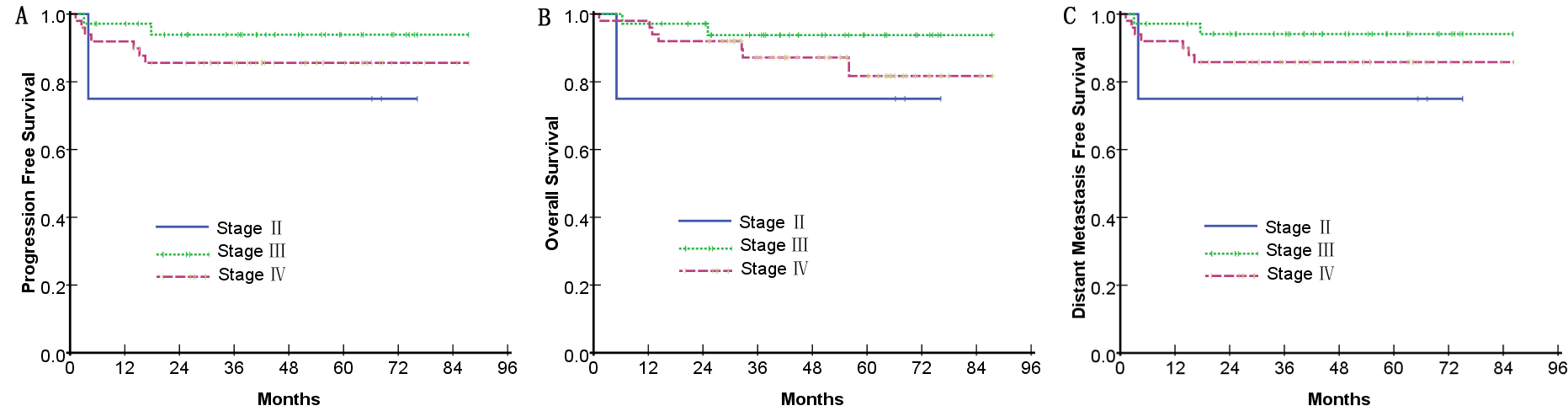

Supplement: Supplemental Digital Content [file medi-94-e1945-s001.doc]
